# Risk Factors and Pathophysiological Mechanisms in Drug-Induced Interstitial Lung Disease Associated With Novel Anticancer Agents: A Scoping Review (2016-2026)

**DOI:** 10.7759/cureus.117422

**Published:** 2026-10-04

**Authors:** Seiichiro Kuroda, Hidetoshi Ishii, Masaaki Ishibashi, Yohei Iimura

**Affiliations:** 1 Department of Pharmacy, The Institute of Medical Science, The University of Tokyo (IMSUT) Hospital, Tokyo, JPN

**Keywords:** antibody-drug conjugates, drug-induced interstitial lung disease, immune checkpoint inhibitors, scoping review, tyrosine kinase inhibitors

## Abstract

Drug-induced interstitial lung disease (DI-ILD) associated with novel anticancer agents, such as immune checkpoint inhibitors (ICIs), tyrosine kinase inhibitors (TKIs), and antibody-drug conjugates (ADCs), is a severe adverse event that can lead to fatal outcomes. Effective clinical management requires integrating host-specific risk factors with drug-specific biological mechanisms. This scoping review, conducted following the PRISMA Extension for Scoping Reviews (PRISMA-ScR) guidelines, systematically searched PubMed and Ichushi-Web databases for peer-reviewed articles published between January 2016 and April 2026. Data from 65 included articles were synthesized into a conceptual framework. Pre-existing ILD or subclinical interstitial lung abnormalities (ILA) emerged as highly prevalent and potent class-transcending risk factors, particularly across novel anticancer drug classes. Distinct temporal patterns of injury were identified: TKIs typically induced DI-ILD within the first one to two months via diffuse alveolar damage (DAD) resulting from epithelial repair blockade, whereas ICI-induced pneumonitis peaked between two and four months due to a breakdown of self-tolerance. To transition from reactive drug interruption to a proactive "Precision Clinical Safety" strategy, we propose a risk-adaptive monitoring protocol centered on mandatory pre-treatment screening with thin-section high-resolution computed tomography (HRCT) to detect subclinical ILA. While low-to-intermediate-risk patients follow standard protocols, high-risk patients require enhanced surveillance, including clinical consultations every two weeks during the initial treatment phase.

## Introduction and background

Drug-induced interstitial lung disease (DI-ILD) represents a severe, non-infectious parenchymal lung disorder resulting from adverse drug exposure, characterized by diffuse pulmonary inflammation and progressive fibrosis that can rapidly lead to fatal respiratory failure. The therapeutic landscape of clinical oncology has been revolutionized by novel agents, including ICIs-monoclonal antibodies that block inhibitory pathways (such as PD-1/PD-L1 and CTLA-4) to reactivate host T-cell anti-tumor immunity-and tyrosine kinase inhibitors (TKIs), which are small-molecule targeted therapies that inhibit specific oncogenic signaling cascades (such as EGFR and ALK). While these advanced modalities have substantially improved patient survival, they have also broadened the clinical spectrum and complexity of DI-ILD [1-5]. 

Notably, Japanese populations consistently exhibit a higher reported incidence and greater clinical severity of DI-ILD compared to Western cohorts. This regional and ethnic disparity is recognized as a multifactorial phenomenon driven by genetic susceptibility (such as specific HLA allele variations and metabolic enzyme polymorphisms), widespread routine baseline screening with high-resolution computed tomography (HRCT) in Japanese clinical practice, and distinctive drug administration profiles [6-10].

Although previous literature reviews have addressed individual aspects of pulmonary toxicity, a critical knowledge gap remains: existing reviews typically focus on isolated pharmaceutical classes (e.g., ICIs alone or EGFR-TKIs alone) or separate clinical epidemiology from basic biological research. Consequently, a comprehensive synthesis that bridges shared versus drug-specific pathophysiological mechanisms (e.g., T-cell self-tolerance breakdown versus epithelial repair blockade) with clinical risk profiles across global and high-incidence Japanese populations is lacking [11-15]. 

In this study, we addressed this gap by formulating our primary research question using the Population, Concept, Context (PCC) framework recommended for scoping reviews: "What are the clinical risk factors (Population/Context) and biological pathophysiological mechanisms (Concept) associated with DI-ILD caused by novel anticancer therapies (ICIs, TKIs, and ADCs) reported in global and Japanese literature between 2016 and 2026 [3,4,6-16].

Conducted in strict accordance with the Preferred Reporting Items for Systematic Reviews and Meta-Analyses extension for Scoping Reviews (PRISMA-ScR) guidelines, this scoping review methodology is specifically justified because our primary objective is to map the broad, heterogeneous evidence base and synthesize conceptual frameworks across diverse study designs rather than compute pooled statistical effect estimates [16-19].

## Review

Materials and methods

Study Design and Guidelines

This scoping review was conducted in strict accordance with the PRISMA-ScR guidelines [20]. In line with the Joanna Briggs Institute (JBI) methodology for scoping reviews [21], the primary research question was formulated using the PCC framework. Population/context: patients receiving novel anticancer therapies (immune checkpoint inhibitors (ICIs), TKIs, or antibody-drug conjugates (ADCs)) reported in global and Japanese clinical literature published between 2016 and 2026. Concept: clinical risk factors and biological pathophysiological mechanisms underlying DI-ILD.

Protocol registration in PROSPERO was not performed, as PROSPERO explicitly excludes scoping reviews from its registration scope, restricting eligibility solely to systematic reviews with health-related outcomes.

Information Sources and Search Strategy

Comprehensive literature searches were conducted across two primary biomedical databases, that is, PubMed/MEDLINE (for international literature) and Ichushi-Web (a domestic Japanese database managed by the Japan Medical Abstracts Society; https://search.jamas.or.jp/). Ichushi-Web was intentionally included to capture indispensable population-specific evidence in Japan, where DI-ILD exhibits a recognized high incidence and severity.

The search strategy utilized a combination of Medical Subject Headings (MeSH) terms and free-text keywords linked with Boolean operators (AND/OR). Key search terms included: ("Drug-Induced Interstitial Lung Disease" OR "Pneumonitis" OR "Drug-Induced Lung Injury" OR "Pulmonary Toxicity") AND ("Immune Checkpoint Inhibitors" OR "PD-1" OR "PD-L1" OR "CTLA-4" OR "Tyrosine Kinase Inhibitors" OR "EGFR-TKI" OR "ALK-TKI" OR "Antibody-Drug Conjugates"). Additionally, supplementary manual reference searches (snowballing) of identified articles were conducted to ensure literature saturation.

Data Charting, Quality Assessment, and Synthesis

Data extraction was performed independently by two reviewers using a standardized data charting form. Extracted variables included study design, patient sample size, drug class, identified clinical risk factors, proposed biological mechanisms, and reported clinical outcomes.

Selection of Sources of Evidence

The initial search yielded 276 articles (PubMed, n = 172; Ichushi-Web, n = 104). After the removal of 12 duplicate records, 264 articles underwent primary screening based on their titles and abstracts. This led to the exclusion of 108 records, including case reports without a comprehensive discussion or reports focusing on non-pulmonary toxicities. The remaining 156 articles underwent full-text eligibility assessment. Following a detailed review of their alignment with the contemporary anticancer agent inclusion criterion and the specificity of descriptive details, 65 studies were ultimately included [1-19,22-67].

Data Charting Process and Data Items

Data were systematically extracted from 65 included studies and were organized into the following categories:

Methodological classification: Studies were categorized into “Group 1 (Mechanism)” focusing on the pathophysiological processes (n = 23) [1,5,6,12,14,18,19,22,23,25,27,31-42], “Group 2 (Risk Factors)” focusing on epidemiological analysis (n = 34) [2,4,8,10,15,16,24,26,28,43-67], or “Both” (n = 8) [3,7,9,11,13,17,29,30].

Data items: The information extracted included drug classes (ICIs, TKIs, and ADCs), clinical risk factors (age, smoking history, pre-existing ILD/interstitial lung abnormalities (ILA)), sex, laboratory parameters including renal function (estimated glomerular filtration rate (eGFR)) and inflammatory markers (Krebs von den Lungen-6 (KL-6), lactate dehydrogenase (LDH), C-reactive protein (CRP)), and performance status), and biological mechanisms. These items were synthesized into a comprehensive evidence table (Table 1).

**Table 1:** Comprehensive Data Charting and Methodological Classification of Included Studies on Drug-Induced Interstitial Lung Disease (DI-ILD) (2016-2026) Studies are stratified into two distinct primary categories. Category 1 (primary focus) comprises studies evaluating novel targeted and immune anticancer therapies (immune checkpoint inhibitors (ICIs), tyrosine kinase inhibitors (TKIs), and antibody-drug conjugates (ADCs)). Category 2 (comparative reference) includes classical non-oncologic or traditional cytotoxic agents serving as a historical baseline for drug-induced pulmonary injury mechanisms. Pre-existing interstitial lung disease (ILD)/interstitial lung abnormalities (ILA) are identified as class-transcending risk factors predominant in Category 1. EGFR-TKI: epidermal growth factor receptor-tyrosine kinase inhibitor; CAR-T: chimeric antigen receptor-T cell therapy; MEK: mitogen-activated protein kinase; ALK-TKIs: anaplastic lymphoma kinase-tyrosine kinase inhibitors; mTOR: mechanistic target of rapamycin; PI3K: phosphoinositide 3-kinase; DMARDs: disease-modifying antirheumatic drugs; MTX: methotrexate; LMWH: low-molecular-weight heparin; PZA: pyrazinamide; INH: isoniazid; RFP: rifampicin; TNFi: tumor necrosis factor inhibitors; 5-ASA: 5-aminosalicylic acid; FDC-HRZE: fixed-dose combination of isoniazid, rifampicin, pyrazinamide, and ethambutol; DOACs: direct oral anticoagulants; CT: computed tomography; HBV: hepatitis B virus; BALF: bronchoalveolar lavage fluid; SP-D: serum surfactant protein; TM: thrombomodulin; PaO_2_/FiO_2_: partial pressure of arterial oxygen to the fraction of inspired oxygen; eGFR: estimated glomerular filtration rate; KL-6: Krebs von den Lungen-6; BMI: body mass index; SGOT: serum glutamic pyruvic transaminase; SGPT: serum glutamic oxaloacetic transaminase; TBM: tuberculous meningitis; IL: interleukin; CIP: checkpoint inhibitor pneumonitis; ATT: anti-tubercular therapy; CNS: central nervous system; ROS: reactive oxygen species; PPIX: protoporphyrin IX; TNF: tumor necrosis factor

No.	Study ID	First author	Year	Drug class/category	Identified host risk factors	Proposed pathophysiological mechanisms	Methodological classification	Reference no.
Category 1: primary focus - novel anticancer agents (ICIs, TKIs, and ADCs)								
1	42047678	Zhu Y	2026	Novel anticancer agents (targeted, ICI, ADC, etc.)	Older age, male sex, pre-existing ILD, concomitant use of pneumotoxic drugs	Class-specific signals detected (e.g., pembrolizumab, durvalumab, osimertinib)	Both	3
2	39379903	Karayama M	2024	ICIs	Organizing pneumonia (OP) pattern on imaging, onset >100 days after ICI initiation	Mechanisms related to relapse after corticosteroid therapy	Both	29
3	35044093	Yamaguchi T	2022	ICI + chemotherapy	Smoking history, reduced lung function, specific treatment regimens	Inflammatory cell infiltration as an irAE due to immune system activation	Both	7
4	33078531	Moda M	2020	ICIs	Tumor invasion into the central airway	Early-onset CIP associated with central airway lesions	Both	30
5	31446020	Fukihara J	2019	PD-1 inhibitors	Smoking history, pre-existing ILD, previous thoracic radiation	Breakdown of self-tolerance and excessive host autoimmune response	Both	9
6	30429014	Cho JY	2018	ICIs	Male sex, older age, smoking history, pre-existing respiratory disease, previous radiation	Lung tissue injury (irAE) via T-cell activation and cytokine release	Both	11
7	2022351858	Sumii M	2022	Targeted agents (EGFR-TKI: osimertinib)	Pre-existing ILA (especially subpleural fibrotic type)	EGFR inhibition leading to reduced repair capacity of alveolar epithelium	Both	13
8	2020226417	Kodama H	2020	ICIs	Male sex, older age (>60 years), smoking, pre-existing ILD, prior lung surgery/radiation	T-cell activation via checkpoint inhibition and inflammatory infiltration	Both	17
9	41484545	Poveda JJR	2026	ICIs, CAR-T, biologics	Pre-existing lung disease (ILD, etc.), older age, smoking history	Direct cytotoxicity to lung cells or disruption of pulmonary immune homeostasis	Group 1	1
10	40768795	Karayama M	2025	ICIs	Pre-existing interstitial lung disease (ILD)	Breakdown of self-tolerance and excessive host autoimmune response	Group 1	5
11	38269311	Nakamura E	2024	Targeted agents (BRAF/MEK inhibitors)	Pre-existing ILD, prior thoracic radiation, pre-treatment fibrotic changes on CT	Inhibition of lung injury recovery processes and induction of damage	Group 1	33
12	37103090	Manabe T	2023	ICIs (with analgesics)	Male sex, older age ≥70 years, concomitant use of opioid analgesics	Additive effects of opioids on immune regulation and cytokine responses	Group 1	22
13	37089604	Sweis JJG	2023	Immune-mediated agents (ICIs, etc.)	Pre-existing autoimmune disease	Enhancement of autoimmune response and cytokine release via checkpoint inhibition	Group 1	34
14	35656938	Zhao YP	2022	Targeted agents (EGFR/ALK-TKIs)	Specific driver gene mutation, Japanese ethnicity (racial factors)	Direct toxicity to alveolar cells or immune-mediated lung injury	Group 1	23
15	35325169	Ohe Y	2022	ICI (atezolizumab)	Male sex, pre-existing interstitial pneumonia	Inflammatory cell infiltration into interstitial tissue following immune activation	Group 1	6
16	33450293	Johkoh T	2021	Targeted agents, ICIs	Temporal correlation with drug exposure (time since initiation)	Direct damage to alveolar epithelium/capillary endothelium or immune disruption	Group 1	38
17	32880733	Shannon VR	2020	ICIs	Pre-existing autoimmune disease, pre-existing lung disease	Loss of self-tolerance and attack on normal tissue (lung) via T-cell activation	Group 1	19
18	32713811	Sakamoto K	2020	ICIs	Respiratory comorbidities	Diverse inflammatory responses involving parenchyma, airways, pleura, and vessels	Group 1	18
19	31852459	Kato M	2019	Idiopathic interstitial pneumonia	High serum KL-6 levels	Acute exacerbation of pre-existing ILD triggered by infectious or non-infectious factors	Group 1	25
20	28212992	Kato T	2017	ICIs	Male sex, smoking history, pre-existing ILD	Breakdown of self-tolerance and lung tissue attack by effector T-cells	Group 1	12
21	2016035934	Yamada S	2015	Monoclonal antibodies (cetuximab)	p16 expression, EGFRvIII expression, severe adverse events	EGFR signaling blockade and cetuximab-induced severe tissue toxicity	Group 1	41
22	2015149643	Yamada S	2014	Monoclonal antibodies (cetuximab)	Recurrent/advanced oral cancer, severe adverse events	Cetuximab-mediated EGFR inhibition and overlapping cytotoxicity	Group 1	42
23	41988621	De S	2026	Targeted agents (TKIs)	Pre-existing ILD, older age, male sex	Direct alveolar injury or immune-mediated response	Group 2	4
24	40892200	Shah DR	2025	Targeted agents (EGFR/ALK/mTOR inhibitors)	Drug class (especially mTOR inhibitors), specific treatment indications	Signal detection (disproportionality analysis) in spontaneous reporting databases	Group 2	47
25	40007198	Hwang IH	2025	Antineoplastic agents (mTORi, ADC, ICI, etc.)	Male sex, older age, pre-existing lung disease, lung cancer patients, mTORi use	Effects on alveolar epithelium associated with mTOR pathway inhibition	Group 2	2
26	32528682	Matsumoto K	2020	Targeted agents, ICIs, amiodarone, Sho-saiko-to	Advanced age (≥50 years), concomitant amiodarone or Sho-saiko-to	Synergistic toxicity and age-related impairment of drug clearance/lung defense	Group 2	15
27	32237210	Koshikawa K	2020	ALK-TKIs	Older age ≥64 years, creatinine clearance <80 mL/min	Lung tissue damage primarily presenting an organizing pneumonia (OP) pattern	Group 2	8
28	31501888	Mosedale M	2019	PI3K inhibitor (Idelalisib)	Genetic susceptibility (QTLs on Chr 17/8 involving bile acid/immune genes)	Altered drug/bile acid clearance and immune-mediated tissue toxicity	Group 2	61
Category 2: Comparative reference - non-oncologic and classical agents								
1	40423461	Kaye AD	2025	Nitrofurantoin (antibiotic)	Chronic exposure, drug hypersensitivity	Oxidative stress by ROS generated during metabolism and immune activation	Group 1	31
2	40282506	Abou Hjeily B	2025	conventional DMARDs (MTX, etc.)	Older age, current smoking, high disease activity, severe pre-existing ILD	Progression from inflammation to fibrosis in the interstitium (e.g., adenosine pathway)	Group 1	32
3	37822623	Fukuda K	2023	Chemo-immunotherapy	Presence and extent of ILA (e.g., >15% of lung field)	Immune-mediated lung tissue damage by agents like ICIs	Group 1	14
4	36569862	Huang Q	2022	Anti-tuberculosis drugs	Genetic polymorphisms in WNT signaling (WIF-1, LRP5, WNT3A, etc.)	Induction of inflammatory response or liver injury based on genetic susceptibility	Group 1	35
5	36483566	Li HM	2022	Anti-tuberculosis drugs	Th17 pathway gene polymorphisms (IL-17A, IL-17F, etc.)	Involvement in drug resistance or inflammatory side effects via specific cytokine pathways	Group 1	36
6	33882819	Zhang TP	2021	Anti-tuberculosis drugs	Vitamin D pathway gene polymorphisms (GC, DHCR7, etc.)	Changes in drug response and safety due to polymorphisms in metabolic enzymes/receptors	Group 1	37
7	32639644	Yang X	2020	LMWH, fondaparinux	Prolonged administration (>14 days), HBsAg positive, diabetes	Leakage of cell contents (e.g., miR-122) via direct action on hepatocyte membranes	Group 1	39
8	30808322	Shirasawa M	2019	Cytotoxic chemotherapy	Comorbid pre-existing interstitial pneumonia	Direct damage to alveolar epithelial cells due to drug cytotoxicity	Group 1	40
9	30029601	Wu S	2018	Targeted agents (crizotinib)	None specified (case report)	Acute lung injury (DAD pattern) due to drug cytotoxicity	Group 1	27
10	41725869	Ishikawa K	2025	Diazoxide	Trisomy 13, low birth weight	Increased right ventricular preload and pulmonary arterial medial defects inducing PH	Group 2	43
11	41694885	Chan ACY	2026	SSRIs	Cardiovascular disease, smoking, chronic kidney disease	Serotonergic valvular interstitial cell proliferation leading to valvular incompetence	Group 2	44
12	41675318	Ou X	2026	Antifibrotics (pirfenidone, nintedanib)	Multimorbidity (≥4), liver disease, cardiovascular disease, drinking	Hepatic metabolic overload and idiosyncratic/direct hepatotoxicity	Group 2	45
13	41639788	Ye D	2026	Anti-tuberculosis drugs	Younger age (<5 yrs), TBM stage III, coma, hydrocephalus	DILI during intensive ATT phase and CNS inflammation/ischemia	Group 2	46
14	40642720	Manokaran V	2025	Anti-tuberculosis drugs	Malnutrition, female sex, elevated baseline SGOT/SGPT	Immaturity of hepatic enzymes and depleted glutathione stores in malnutrition	Group 2	48
15	40491731	Semwal P	2025	Anti-tuberculosis drugs (PZA, INH, RFP)	Younger age, female sex, malnutrition, hypoalbuminemia, NAT2/CYP2E1 polymorphisms	Accumulation of toxic metabolites, ROS generation, and HLA-linked hypersensitivity	Group 2	49
16	39816129	Ilham AF	2024	Anti-tuberculosis drugs	Viral hepatitis co-infection (HBV/HCV), older age, alcohol consumption	Impaired hepatic clearance and synergistic hepatotoxicity with viral hepatitis	Group 2	50
17	39482644	Cartlidge MK	2024	Rheumatological DMARDs (MTX, TNFi, etc.)	Older age (≥70 yrs), current smoking, high disease activity, UIP/honeycombing, progressive ILD	Immune perturbation in a pre-injured fibrotic/inflammatory lung microenvironment	Group 2	51
18	38699076	Feng Y	2024	Anti-tuberculosis drugs	DILI, acute heart failure, chronic kidney disease, extrapulmonary TB	Systemic organ toxicity and adverse drug events complicating anti-tuberculosis therapy	Group 2	52
19	38131189	Nishijima S	2024	Breast cancer agents (targeted, cytotoxic)	Use of ADCs, CDK4/6 inhibitors, or mTOR inhibitors	Pulmonary toxicity patterns of specific regimens in Japanese patients	Group 2	53
20	35691329	Moda M	2022	5-ASA/mesalamine, biologics	Active ulcerative colitis, male sex, prior mesalamine exposure	Drug-induced organizing pneumonia (OP) or extraintestinal immune-mediated lung injury	Group 2	54
21	35629211	Cavaco MJ	2022	Anti-tuberculosis drugs	NAT2 slow acetylator (SA) phenotype, older age ≥55 years	Accumulation of toxic metabolites due to genetically reduced acetylation capacity	Group 2	28
22	35345618	Yoshioka K	2022	nab-Paclitaxel	Male sex, older age ≥56 years, pancreatic cancer, pre-existing ILA, use with GEM	Inflammatory response in lung tissue induced by cytokine production	Group 2	55
23	35129672	Kaku S	2022	Antineoplastic drugs (cytotoxic, targeted, ICI)	Pre-existing ILD, poor PS (2-4), DAD imaging pattern	Severe parenchymal damage and impaired alveolar repair following drug administration	Group 2	56
24	34819852	Zhong T	2021	Anti-tuberculosis drugs (FDC-HRZE, PZA)	Excessive PZA dose, longer treatment duration (>30 days), 4FDC regimen	Hepatic toxin accumulation (PPIX/bile acids) due to INH/RFP/PZA metabolic load	Group 2	57
25	34684131	Ugajin M	2021	Anticoagulants (DOACs, warfarin, heparin)	Anticoagulant use, pre-existing diffuse lung disease/ILD, hematuria	Exacerbation of alveolar microvascular bleeding leading to diffuse alveolar hemorrhage	Group 2	58
26	34470841	Cao J	2021	Anti-tuberculosis drugs	Older age, adverse drug reactions (DILI), low BMI	Treatment interruption and organ impairment caused by drug toxicity	Group 2	59
27	33175298	Takeda T	2021	GEM + nab-paclitaxel	ABO blood type (Type B), pre-existing lung disease (emphysematous changes, etc.)	Genetic susceptibility associated with specific blood type antigens	Group 2	16
28	32669522	Ali N	2020	Anti-tuberculosis drugs	Malnutrition, low serum albumin, low BMI	Depletion of hepatic glutathione and altered drug clearance in malnourished states	Group 2	60
29	30521972	Gemma A	2019	Crizotinib	Pre-existing ILD, poor performance status (PS ≥2), male sex	Direct damage and repair inhibition of alveolar epithelial cells	Group 2	10
30	30381644	Sakurada T	2018	Cytotoxic and targeted anticancer agents	Older age, renal/hepatic dysfunction, prior lung disease, combination therapy	Increased systemic drug exposure and reduced physiological repair capacity	Group 2	62
31	30118797	Yorifuji K	2018	Endothelin receptor antagonist (bosentan)	CHST3 and CHST13 gene polymorphisms, older age, elevated serum creatinine	Altered glycosaminoglycan sulfation and BSEP inhibition leading to hepatotoxicity	Group 2	63
32	29413509	Abe M	2018	Everolimus	High pre-treatment serum KL-6, reduced eGFR	Inhibition of lung injury recovery process via mTOR pathway suppression	Group 2	24
33	27976836	Suzuki A	2017	Antineoplastic, antithrombotic, amiodarone	High BALF soluble TM, elevated serum SP-D, low PaO2/FiO2 ratio (<300)	Pulmonary endothelial cell damage and acute alveolar-capillary barrier disruption	Group 2	64
34	27832142	Van't Boveneind-Vrubleuskaya N	2016	Anti-tuberculosis drugs	Development of DILI, initial lung cavitation, baseline high bacterial load	Treatment interruption and dose modifications necessitated by DILI	Group 2	65
35	26935778	Sun Q	2016	First-line anti-tuberculosis drugs (PZA, INH, RFP)	HBV carrier status (HBsAg+), older age, malnutrition, extensive lung lesions	Synergistic liver injury from viral hepatitis replication and toxic ATT metabolites	Group 2	66
36	2012241627	Nakamichi H	2012	Gemcitabine (GEM)	Male sex, older age ≥65 years, smoking history, first-line treatment	Mixture of dose-independent allergic reactions and direct lung injury	Group 2	67
37	2009253617	Tsumura T	2009	Gemcitabine (GEM)	Pre-existing lung lesions on CT (emphysema, inflammation from metastases, etc.)	Induction of TNF-α, IL-1α, IL-6, etc., at inflammatory sites	Group 2	26

In accordance with official PRISMA-ScR guidelines (Item 12) and JBI scoping review standards, critical appraisal (risk of bias assessment) of individual studies was omitted. The principal objective of a scoping review is to comprehensively map the breadth of evidence, synthesize biological mechanisms, and establish a conceptual framework across highly heterogeneous study designs, rather than evaluating individual study quality to estimate a pooled effect size. For the same reason, quantitative statistical meta-analysis was neither methodologically feasible nor appropriate due to severe clinical and mechanistic heterogeneity. Instead, findings were synthesized qualitatively into a structured evidence matrix and an integrative conceptual framework.

Synthesis of Results

The extracted data were analyzed quantitatively to map the frequency of the reported DI-ILD risk factors across the different drug classes and were visually depicted using a bubble chart. For qualitative synthesis, we constructed a conceptual framework centered on three primary pathophysiological scenarios to structure the interactions between specific host risk factors and pharmacological triggers in producing clinical DI-ILD phenotypes.

Results

Selection of Sources of Evidence

The systematic literature selection process is summarized in a PRISMA flow diagram (Figure 1). The initial search yielded 172 records from PubMed and 104 from Ichushi-Web, totaling 276 records. After removing 12 duplicate records, titles and abstracts of 264 articles were screened. During this phase, 108 articles were excluded, which were primarily case reports lacking a comprehensive discussion or studies focusing on toxicities other than DI-ILD. The remaining 156 articles underwent a full-text assessment for eligibility. Out of these, 91 were excluded because they did not involve contemporary anticancer agents or included insufficient descriptive detail. Ultimately, 65 studies (59 international and 6 domestic Japanese articles) were included in this review [1-19,21-67]. These articles were identified through systematic literature searches across international and domestic databases, including Ichushi-Web [68]. These consisted of 23 studies in “Group 1 (mechanism)” [1,5,6,12,14,18,19,22,23,25,27,31-42], 34 in “Group 2 (risk factors)” [2,4,8,10,15,16,24,26,28,43-67], and 8 in “Both” [3,7,9,11,13,17,29,30].

**Figure 1:**
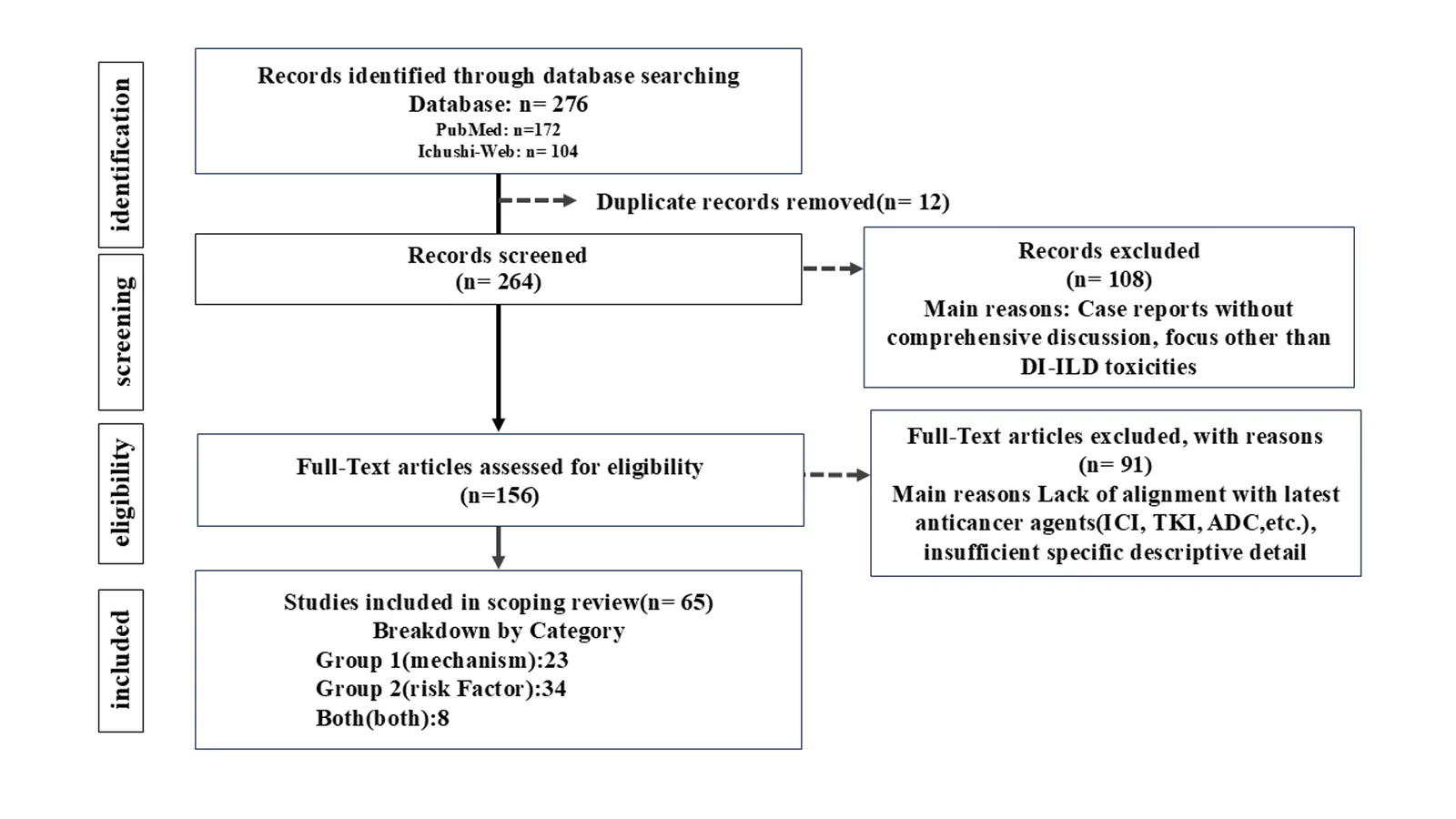
PRISMA Flow Diagram Demonstrating Literature Selection and Inclusion Process The diagram outlines the identification, screening, eligibility, and inclusion process of 65 studies in accordance with the PRISMA Extension for Scoping Reviews (PRISMA-ScR) guidelines. From an initial pool of 276 records identified through PubMed (n = 172) and Ichushi-Web (n = 104), 65 studies met the final eligibility criteria. The included studies were categorized into three groups: those in group 1 focused on biological mechanisms (n = 23) [1,5,6,12,14,18-21,23,25,29-40], those in group 2 focused on clinical risk factor analysis (n = 34) [2,4,8,10,15,16,22,24,26,41-65], and those in group 3 addressed both aspects (n = 8) [3,7,9,11,13,17,27,28].

Characteristics of Sources of Evidence

The distribution of evidence across the 65 included studies is summarized in Table 1. Reports on ICIs were most frequent, followed by those on TKIs and ADCs. Study designs primarily included pharmacovigilance analyses using large-scale spontaneous reporting databases (e.g., FAERS and JADER), retrospective single- or multicenter cohort studies, and narrative reviews synthesizing the latest findings. Studies categorized as “Both” often linked clinical risk factors to biological mechanisms through large-database analyses. Also, HRCT was utilized in most studies as the standard imaging tool for evaluating pre-existing ILA and facilitating early diagnosis of DI-ILD.

Mapping of Risk Factors Across Drug Groups

An analysis of correlations between specific drug classes and reported host-related risk factors revealed distinct frequency patterns unique to each class (Figure 2) [1-13,19,22]. Although this scoping review primarily focuses on novel anticancer agents, clinical studies involving anti-tuberculosis drugs, methotrexate, and amiodarone were strategically included in the search criteria. These non-oncology therapeutics serve as vital positive controls and reference standards to provide robust evidence regarding shared, class-independent pathways of pulmonary injury, such as genetic polymorphisms in drug-metabolizing enzymes or localized oxidative stress. By evaluating these comparative references alongside modern oncological agents, we identified several critical class-specific and class-transcending trends in the clinical susceptibility to DI-ILD.

**Figure 2:**
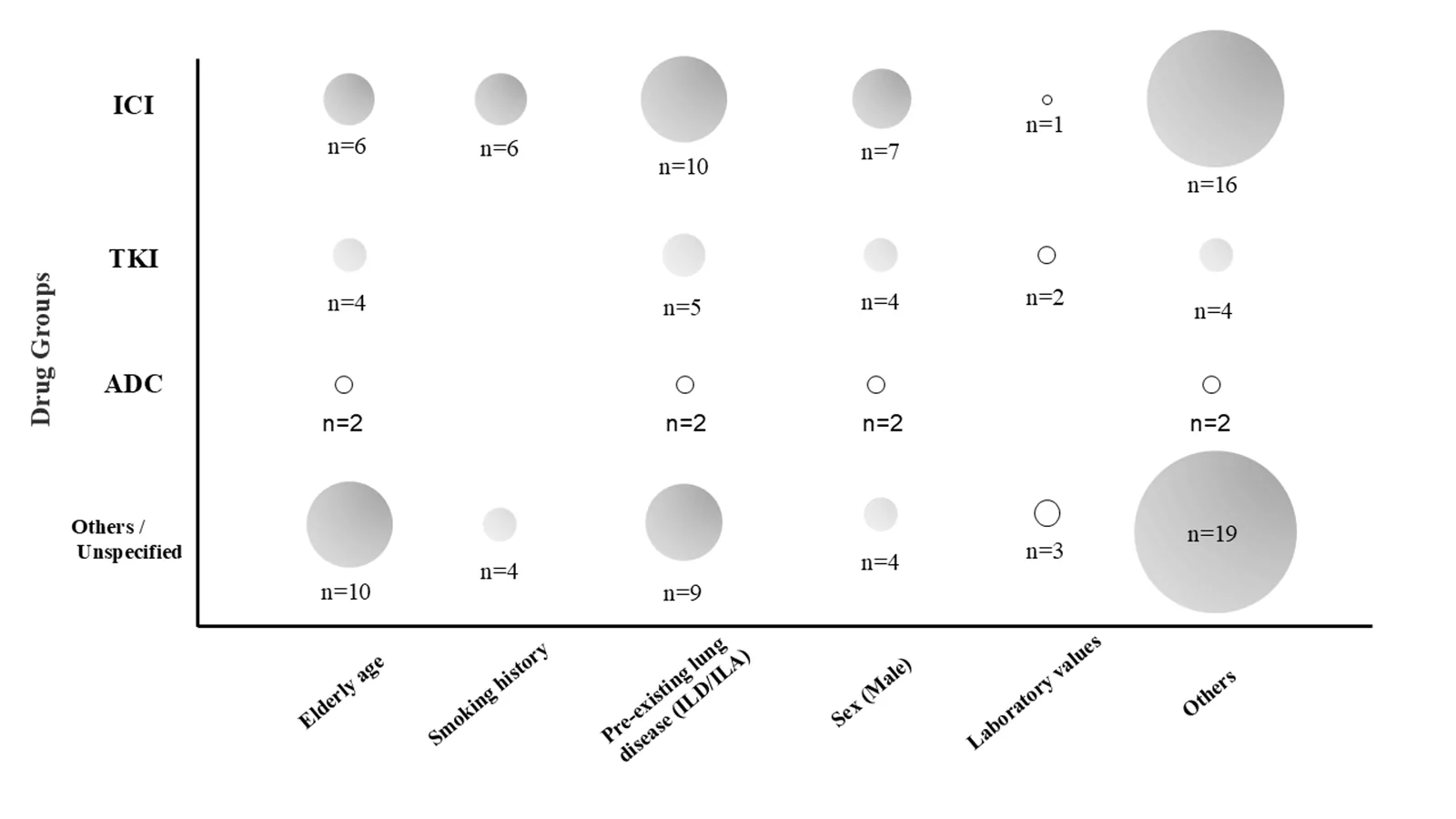
Mapping of Clinical Risk Factors Across Major Anticancer Drug Classes (2016-2026) The bubble chart visually depicts the distribution and frequency of clinical risk factors associated with immune checkpoint inhibitors (ICIs), tyrosine kinase inhibitors (TKIs), and antibody-drug conjugates (ADCs) identified over a 10-year period (Jan 2016-Apr 2026). Bubble size corresponds to the occurrence frequency (number of evidence sources) identifying each factor. Pre-existing interstitial lung disease (ILD) or subclinical interstitial lung abnormalities (ILA) is highlighted as a high-potency, class-transcending risk factor across novel anticancer therapies, whereas factors such as older age and smoking history exhibit class-specific prevalence. “Others” include genetic polymorphisms, specific blood types, and previous radiation therapy.

For ICIs, "pre-existing lung disease" (primarily ILD or subclinical ILA) was identified as the most dominant clinical risk factor, appearing in ten independent studies [3,5-7,9,11,13,17,19,29]. This strong association is pathophysiologically logical; the baseline inflammatory microenvironment and structural lung damage in patients with pre-existing pulmonary lesions likely lower the threshold for severe immune-related adverse events. When ICIs block T-cell inhibitory pathways, this compromised pulmonary microenvironment becomes highly susceptible to an accelerated breakdown of self-tolerance, leading to exaggerated T-cell activation against alveolar self-antigens. Following pre-existing lung lesions, "male sex" (n = 7 studies) [3,6,10-12,17,22], "older age" (n = 6 studies) [6,7,9,11,12,18], and "smoking history" (n = 6 studies) [7,9,11,12,14,22] were identified as key risk factors. These demographic features likely represent a synergistic clinical phenotype; older male patients with significant smoking histories frequently harbor subclinical, chronic alveolar remodeling or emphysema, which exacerbates inflammatory susceptibility during immunotherapy.

Regarding tyrosine kinase inhibitors (TKIs), the clinical susceptibility profile closely mirrors that of ICIs, with "pre-existing lung disease" emerging as the most frequently reported risk factor across five studies [8,10,13,23,24]. The pathological mechanism underlying TKI-induced lung injury is closely tied to the blockade of essential growth factor signaling pathways, such as EGFR or ALK, which are critical for the survival, proliferation, and repair of alveolar epithelial cells (AECs). In patients with baseline lung damage, this signaling blockade prevents the necessary epithelial repair following routine cellular stress, rapidly leading to diffuse alveolar damage (DAD). Demographic factors, including "older age" and "male sex", were also consistently identified as significant risk factors across four studies each [4,8,10,24]. These host factors likely correlate with age-related declines in pulmonary regenerative capacity and a higher prevalence of subclinical environmental or tobacco-related lung changes, both of which markedly impair the lung's capacity to withstand TKI-mediated epithelial toxicity.

For ADCs, clinical data regarding risk factors remain relatively limited due to the novelty of these agents, yet "older age," "pre-existing lung disease," and "male sex" were each identified as prominent risk factors in two clinical studies [2,3]. Unlike pure molecularly targeted therapies or immunotherapies, ADC-induced ILD is hypothesized to involve a dual mechanism of injury, combining direct, dose-dependent cytotoxicity on alveolar macrophages or epithelial cells with secondary immune-mediated inflammatory cascades. Thus, patients with advanced age or pre-existing subclinical lung abnormalities possess a significantly reduced physiological reserve to tolerate the localized, payload-mediated cytotoxic insults of ADCs. As these novel agents gain broader clinical adoption, continuous pharmacovigilance is highly warranted to further elucidate whether these baseline patient characteristics act synergistically to accelerate the onset of fatal pulmonary toxicities.

Among clinical studies categorized as "Others or Unspecified," which predominantly involved conventional cytotoxic chemotherapies and anti-tuberculosis regimens, "older age" (n = 10 studies) [3,4,16,25-28] and "pre-existing lung disease" (n = 9 studies) [3,4,16,25-27] were highlighted as crucial determinants of severe drug-induced pulmonary impairment [28,31,32,35-40,43-46,48-52,54,57-67]. In these traditional treatment settings, advanced age is strongly associated with a progressive decline in hepatic and renal clearance functions, leading to systemic drug accumulation and heightened susceptibility to direct tissue toxicity. Ultimately, regardless of the therapeutic class administered. Pre-existing ILD or subclinical abnormalities represent a highly potent, class-transcending driver of drug-induced pulmonary toxicity, demanding rigorous clinical surveillance and proactive risk-mitigation strategies across all treatment modalities.

Key trends in biological mechanisms

Integration of the extracted evidence identified three primary pathophysiological trends involved in DI-ILD onset, which were structured into a conceptual framework (Figure 3) [1-19,22-67].

**Figure 3:**
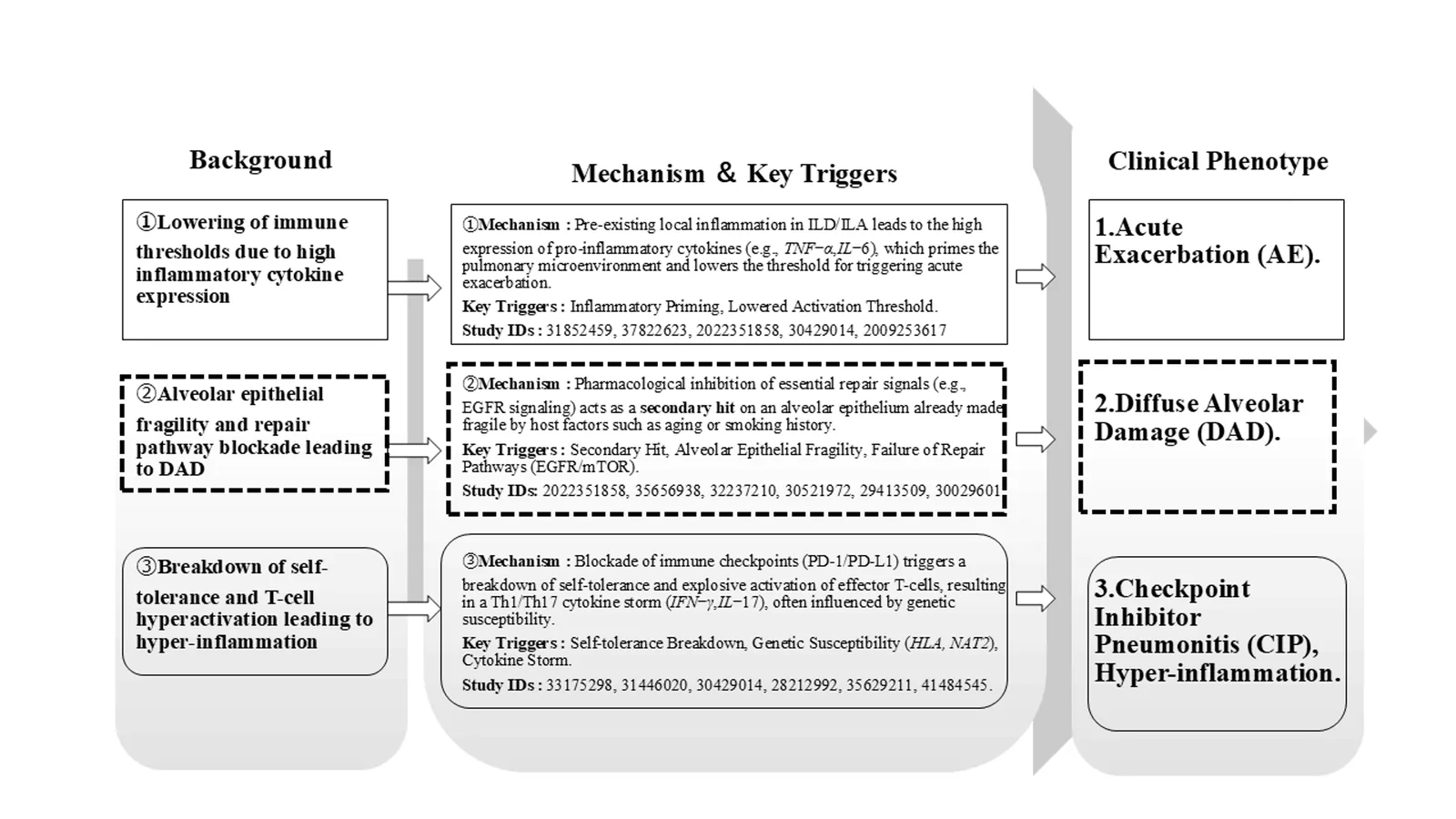
Integrated Conceptual Framework of Pathophysiological Mechanisms in Drug-Induced Interstitial Lung Disease This framework illustrates the synergistic relationship between host-specific risk factors and pharmacological triggers across three primary pathophysiological scenarios. This conceptual mapping illustrates three major injury pathways. Solid lines denote established pathways (e.g., TKI-mediated epithelial repair inhibition); dashed lines denote proposed/hypothesis-generating pathways (e.g., ICI autoantigen recognition and ADC payload bystander toxicity).

Scenario 1: Lowering of Immune Thresholds Due to High Inflammatory Cytokine Expression

Pre-existing local inflammation and/or metabolic/genetic factors lead to high expression of pro-inflammatory cytokines (e.g., tumor necrosis factor (TNF)-α, interleukin (IL)-6), which primes the pulmonary microenvironment and lowers the activation threshold for triggering acute exacerbations or severe systemic organ toxicities [2,15,16,22,25,26,28,31,32,35-37,39,43-46,48-50,52,55,57-61,63-67].

Scenario 2: Alveolar Epithelial Fragility and Repair Pathway Blockade Leading to DAD

Pharmacological inhibition of essential cellular repair pathways (e.g., epidermal growth factor receptor (EGFR), anaplastic lymphoma kinase (ALK), or mechanistic target of rapamycin (mTOR) signaling) acts as a "secondary hit" on an alveolar epithelium already made fragile by host-specific risk factors such as advanced age or preexisting interstitial abnormalities, resulting in DAD or severe parenchymal toxicity [1-4,8,10,13,23,24,27,33,38,40-42,47,51,53,56,62].

Scenario 3: Breakdown of Self-Tolerance and T-cell Hyperactivation Leading to Hyper-Inflammation

Blockade of immune checkpoint pathways (PD-1/PD-L1 or CTLA-4) triggers a structural breakdown of self-tolerance and explosive, uninhibited activation of effector T-cells, culminating in a Th1/Th17 cytokine storm and immune-related adverse events (irAEs) across the lung parenchyma, airways, and vascular endothelium [1,3,5-7,9,11,12,14,17-19,29,30,34,54].

Discussion

Correlation Between Risk Factors and Biological Mechanisms

Synthesis of the included 65 studies demonstrates a logical bridge connecting host-specific clinical risk factors with drug-induced biological tissue injury mechanisms across four major pathophysiological paradigms.

Pre-existing subclinical ILA/ILD and epithelial fragility/repair blockade (the "two-hit" model): Pre-existing lung abnormalities (such as pre-existing ILD or ILA) represent a highly prevalent, class-transcending clinical risk factor that significantly increases patient vulnerability to severe pulmonary injury, particularly among individuals treated with novel targeted and immune anticancer therapies including ICIs, TKIs, ADCs, and cytotoxic agents [1-11,13,14, 17-19,22-25,29,32-34,38,42,44,47,50,51,53,56,59,62]. Mechanistically, pre-existing fibrotic or inflammatory alterations reduce baseline physiological reserve and create an anatomically fragile alveolar epithelium [7,10,13,23,24,27,33,38,40-42,47,51,53,56,62]. Administration of molecularly targeted agents (e.g., EGFR-TKIs such as osimertinib, ALK-TKIs, mTOR inhibitors, or ADCs) acts as a "secondary hit" by blocking crucial epithelial repair pathways (such as EGFR signaling, ERK/MAPK cascades, or mTOR-mediated cellular proliferation), suppressing mucosal regeneration and driving rapid progression toward DAD or severe organizing pneumonia [2-4,8,10,13,23,24,27,33,38,40-42,47,51,53,56,62].

Demographic risk factors: Male sex, older age, smoking and inflammatory priming male sex, advanced age (≥65-70 years), and tobacco smoking history were consistently identified as primary demographic risk factors, particularly in ICI- and TKI-induced pneumonitis cohorts [1,3,5,6,8,9,11,12,15,17,22,29,31,34,42,49,54-57,60,62,66,67]. Aging impairs drug clearance and alveolar type II cell turnover, while chronic smoking causes ongoing bronchial micro-inflammation, elevating baseline pulmonary pro-inflammatory cytokines (e.g., TNF-α, IL-1, IL-6) and vascular endothelium injury markers (such as soluble thrombomodulin, SP-D, and KL-6). This baseline "inflammatory priming" substantially lowers the activation threshold for drug-induced tissue damage, predisposing the lungs to explosive acute exacerbations upon drug exposure [15,16,22,25,26,28,30,32,35-37,39,43--46,48-50,52,55,57-61,63-67].

Immune checkpoint inhibition, autoimmune phenotypes, and breakdown of self-tolerance in immune-checkpoint inhibitor (ICI) regimens, specific clinical features, including central airway tumor invasion, concomitant opioid/analgesic use, previous thoracic radiation, and autoimmune background, correlate strongly with checkpoint inhibitor pneumonitis (CIP) [1,3-7,9,11,12,14,17-19,22,29,30,34,54]. ICIs targeted against PD-1/PD-L1 or CTLA-4 breach immune self-tolerance, triggering uninhibited effector T-cell activation against healthy pulmonary tissue. Local tissue injury from central tumor airway invasion or prior radiation exposes pulmonary neoantigens, which, combined with opioid- or cytokine-mediated immune modulation, accelerates an explosive Th1/Th17 cytokine storm, lymphocytic alveolitis, and organizing pneumonia (OP) patterns.

Host pharmacogenetics, metabolic overload, and organ dysfunction in non-oncologic drug categories: Anti-tuberculosis regimens, anti-rheumatic DMARDs, bosentan, and pirfenidone/nintedanib highlight the profound impact of host pharmacogenetics and organ clearance capacity [11,28,31,32,35-37,39,43-46,48-52,57-61,63,65,66]. Single nucleotide polymorphisms (SNPs) in metabolic and immune pathways - such as NAT2 slow-acetylator phenotypes, CYP2E1 variants, WNT signaling genes (WIF1, LRP5), Th17 cytokine genes (IL-17A/F), vitamin D receptor pathways, and CHST3/13 or BSEP transport genes - directly impair drug metabolism and hepatopulmonary clearance [17,18,23,28,35-37,48,58,61,63]. Concurrent malnutrition, hypoalbuminemia, viral hepatitis co-infection, and renal/hepatic insufficiency deplete glutathione reserves and increase systemic unbound drug exposure, leading to toxic metabolite accumulation, reactive oxygen species (ROS) generation, and severe multi-organ adverse reactions [27,28,37,39,43,45,46,48-50,52,55,57,59,60,65,66].

Drug-Specific Associations and Pathophysiological Interpretation

The distribution of reported frequencies for specific drug-host factor pairs shown in Figure 2 confirmed that DI-ILD onset is determined by the synergy between drug class-specific pharmacology and host factors. The prominent correlation between ICIs and pre-existing lung disease (n = 10) suggests that, in lung tissue, where local self-tolerance mechanisms are exhausted by chronic inflammation, administration of ICIs, which removes the brakes on T-cells, acts as the primary trigger for pathological hyperimmune responses. Conversely, for TKIs, factors such as “older age” and “male sex” were identified as risk factors alongside “pre-existing lung disease” (n = 4 each), suggesting that the pharmacological inhibition of repair signals (e.g., EGFR) acts as a “second-hit” insult on a host with aging-associated physiological decline in alveolar epithelial regeneration (Figure 2).

Proposals for Risk-Adaptive Monitoring Strategies (Clinical Implications)

The conceptual framework (Figure 3) and data synthesis (Figure 2) established in this study provide an evidence-based foundation for developing concrete screening and monitoring strategies. DI-ILD management should be implemented as “Precision Clinical Safety,” integrating host vulnerability with the drug's pharmacological profile.

Optimization of Pretreatment Screening

For patients starting ICIs or TKIs, screening should extend beyond a clinical diagnosis of ILD to evaluate thin-section HRCT scans for subtle GGOs and reticular shadows (indicating ILA). Pretreatment risk stratification should also consider overlapping factors, such as age and smoking history.

Risk-Adaptive Monitoring Frequency

High-risk group: For high-risk patients (e.g., those with preexisting ILD/ILA receiving ICIs), we strongly recommend detailed clinical interviews querying exertional dyspnea and dry cough at every cycle during the first three to four months when DI-ILD risk peaks, along with once or twice monthly serum biomarker measurements (e.g., KL-6 and serum surfactant protein (SP-D)) and periodic chest imaging. For ultra-high-risk cases, close monitoring (via consultations and interviews) every 2 weeks during the initial phase should be considered.

Intermediate to low-risk group: For the intermediate to low-risk group (e.g., for those with only age/smoking risks receiving TKIs), management should focus on patient education for self-monitoring of symptoms, biomarker evaluation every few months, and early detection of epithelial repair failure through strict monitoring of oxygen saturation during routine visits.

Limitations

This scoping review has some limitations.

First, a methodological limitation of this scoping review is the search boundary restricted to Ichushi-Web and PubMed/MEDLINE. While PubMed captured mainstream international literature and Ichushi-Web provided essential access to Japanese population-specific evidence-where DI-ILD incidence is notably elevated-other major databases such as Embase, Web of Science, and Scopus were not searched. Although manual reference snowballing was performed across all 65 included studies to ensure high literature coverage, the exclusion of Embase and Scopus may have led to the omission of certain European multi-center trials or gray literature/conference proceedings, which should be considered when generalizing these findings globally.

Second, per PRISMA-ScR guidelines, our primary objective was to map existing literature and bridge the gap between risk factors and mechanisms, rather than to grade study quality. Consequently, no formal risk of bias or quality assessment was performed on the 65 included articles. Therefore, the findings in our conceptual framework (Figure 3) and evidence table (Table 1) should be interpreted as a comprehensive conceptual overview of the accumulated literature, rather than as findings derived from a statistical pooled analysis or meta-analysis.

Third, significant heterogeneity existed among the included studies regarding cancer types, specific drug regimens, and DI-ILD diagnostic criteria or severity classifications. Relatively few studies provided integrated evidence directly linking specific host factors to precise molecular pathways within a single cohort, which limited our ability to establish definitive relative risks for specific drug-host factor combinations.

## Conclusions

This scoping review synthesizes clinical and mechanistic data from 65 peer-reviewed articles to provide a comprehensive landscape of DI-ILD associated with novel anticancer agents, including ICIs, TKIs, and ADC. Pre-existing ILD or subclinical ILA represents a highly prevalent, class-transcending host risk factor that markedly increases patient susceptibility to severe and fatal pulmonary toxicities, particularly across novel anticancer drug classes. Furthermore, distinct class-specific temporal and pathophysiological patterns characterize each drug category, with tyrosine kinase inhibitors typically inducing acute epithelial impairment within the first two months, and ICIs triggering a breakdown in self-tolerance over a broader onset window of two to four months.

Clinical oncology must transition from reactive drug interruption to a proactive "Precision Clinical Safety" paradigm to mitigate the risk of these fatal toxicities. We propose a risk-adaptive monitoring protocol centered on mandatory pre-treatment screening with thin-section HRCT to detect subclinical lung abnormalities. While low-to-intermediate-risk patients follow standard clinical guidelines, high-risk individuals require enhanced, close-interval surveillance, including scheduled clinical consultations every two weeks during the critical initial phase of treatment. Implementing this prospective, risk-stratified vigilance framework holds the potential to safeguard patients, optimize the therapeutic window of novel oncological agents, and prevent severe adverse outcomes in clinical practice.
